# Massage: A Branch of Physical Treatment

**Published:** 1918-02-02

**Authors:** 


### MASSAGE: A BRANCH OF PHYSICAL TREATMENT.

### Its Principles and its Value.

Among the various forms of treatment applied to the surface of the human body, massage or manipulation has always held an important place. It is well to remember that in point of fact a very large number of movements, some very gentle and others quite the reverse, are comprehended under this general name. Discarding the French nomenclature and using simple English words, massage includes the movements of *touch* and *stroking*; *friction*, *rubbing*, *pinching*, *kneading*, *rolling*, *squeezing* and *wringing*; *vibration*, *tapping*, *clapping*, *hacking*, *beating* and *whipping*; and also *stretching* and *exercising* both of the joints and muscles. The movement or mobilisation of muscles and joints may be purely passive, or may be effected against the muscular resistance of the patient. Most authorities agree that some of these movements, like vibration and whipping, can be better done by mechanical means than by the human hand, and the same is probably true of certain kinds of exercise.

All these various forms of movement, communicated by the hand or otherwise, may be considered as the converse or complement of *rest*. They ought to be employed with alternation and rhythm, like natural movements, which are always alternated with periods of repose. A guiding principle may be found in the fact that energy is needed where natural movement is defective, and often harmful where natural movement is already in excess.

It will be obvious that there are differences of *kind* as well as of *degree* among these many procedures. The regulation of the quantity or dosage of applied movement is sometimes all-important, especially in these days of nervous strain and sensitive wounded limbs. The good effects of massage are by no means in proportion to the amount of energy employed. An excessive or a too-prolonged application defeats its own purpose. Among the chief differences of kind or quality of movement may be mentioned: (1) stroking, friction, or rubbing, in which the hand of the operator *passes over* the surface of the skin; (2) a movement of the skin over the deeper parts, or of one tissue upon another, in which the operator's hand is in contact throughout with the same portion of skin, as in rolling, kneading, and pinching; (3) the blow, including vibration; (4) stretching and exercising movements.

The physiological effect of movements so various must be complex. The body is very sensitive to every application of energy. One feature, however, is common to all of them. The action of surface treatments of whatever kind is twofold:—(1) reflex and (2) local.

The many *reflex effects of massage* are at present insufficiently understood; but they are of great practical importance. Beginning with touch and stroking, powerful sedative effects often result from the gentlest movements. This is familiar in the skilled treatment of insomnia and neurasthenia. Reflex stimulation is equally familiar, as a result of all more energetic movements. In the same manner the *local action of massage* comprises a whole range of sedative and stimulant reactions, which may now be profitably studied in the local treatment of wounded limbs.

In order to a full understanding of the subject it is clear that a comprehensive experimental study ought to be made into the reflex and local actions of all these procedures, and that at present, failing this experimental research, the teaching and practice of massage are to a large extent empirical. There is no lack of books upon massage; but they consist for the most part of anatomical treatises, written for the use of non-medical classes, together with admirable descriptions and illustrations of the several movements that can be effected. This is not quite satisfying to the student of rational treatment. He wants to know all about the agencies which he employs and the effects that they produce on the body, both in health and disease. Something more than special text-books for the masseur are now needed. The remedial value of movement must be considered in the light of modern knowledge. And it is the more desirable now, when many thousands of wounded men in towns and villages stand in immediate need of all remedies that can be properly employed to restore to them health and the use of their limbs.

It is therefore a pleasure to find a book, Dr. J. B. Mennell's "Massage: its Principles and Practice,"* which, like Kellogg's "Art of Massage," attempts to define and disentangle the various procedures that are included under this comprehensive title, and helps to enlighten the prevailing darkness as to the precise place which massage ought to occupy in the great group of physical remedies which are applied, like light, heat, and electricity, to the surface of the body.

We now know that these surface treatments can not only be used with gratifying success in medical war cases, but also assist and supplement the work of the surgeon, and when patiently and scientifically applied will often complete the restoration of wounded limbs. The neglect of these methods by medical men hitherto has led to their unscientific use by irregular practitioners, who by unwise specialisation and extravagant claims have made it difficult to distinguish the true from the false. The war, which has changed so much, will, it is to be hoped, change that also, and give us a new science and art of physical treatment.

It is a remarkable fact that from the remotest times the practice of massage has been associated with applications of heat. The combined method was, or is, constantly in use in the Greek and Roman, Egyptian and Turkish baths, as well as in the

* Published by J. and A. Churchill, with an Introduction by Sir Robert Jones, C.B., F.R.C.S. Pp. xiii., 352, with 135 Illustrations. Price 8s. 6d. net.

**MASSAGE**—(*continued*).

ancient and modern sweating baths of the Russians and other northern nations, not forgetting the Celtic vapour baths at one time prevalent in the British Islands. Mankind everywhere has used heat and moisture as a preparation for massage, because heat and moisture increase the circulation, relieve pain, and facilitate movement. The same principle underlies the latest procedure by which stiffened, wasted, and painful limbs are now treated in physical clinics and hospitals throughout the country. Dr. Mennell is fully alive to the value of a combined physical treatment. Referring to the results of sepsis in a limb and the danger of lighting up infection by vigorous treatment, he points out that repair is made possible by increase of the circulation, and that the whirlpool bath, in which as much as possible of the injured limb is immersed in the water, sometimes hastens recovery in a remarkable manner. Unless a vascular supply adequate for repair is secured, local treatment is useless, and may even be detrimental. Dr. Mennell's experience agrees with that of other recent authorities that the first " free " movement of an injured limb is often best effected *in the bath*, which gives an even support to the part immersed.

The action of manipulation baths, in which massage or movement is applied under water at varying temperatures, is not, however, described in this volume, nor the combined treatment known as the " Massage Douche," which has been successfully employed for many years in dealing with chronic toxic diseases and circulatory disorders, and has made the reputation of more than one health resort.

Dr. Mennell rightly emphasises the many reflex effects of the gentlest forms of massage applied to different areas of the skin. There is, for example, an active gastro-cutaneous reflex. If Sir William Osler is right in enumerating seventeen neuroses of the stomach—motor, sensory, and secretory—there is evidently an ample field for acting upon the stomach through the related skin area. Moreover, it is sometimes better to soothe an overtired horse than to flog him. We are reminded to follow the rhythm of nature, and that stimulation too long continued passes on to paralysis.

In dealing with the applications of massage to surgical conditions, a useful practical direction is given for its employment in recent injuries. To prescribe the due proportion of immobilisation and of movement is sometimes a difficult problem. Another guiding principle to which reference is made is the *right position* for weak or injured parts. A masseur may easily cause serious damage by stretching a weak or paralysed muscle. The *postural splint*, to which the modern school of orthopædic surgeons attaches such prime importance, is designed to keep the affected muscles in a state of relaxation. If this is done, the limb can be safely treated by massage or electricity upon the splint.

As regards the rather vexed question of the value of mechanical apparatus for exercises, Dr. Mennell is an advocate of the pulley-weight machine, as used at Shepherd's Bush, with attached ladder and simple appliances for wrist movements and rotation of the forearm. If these are provided and a nautical wheel and stationary bicycle, he sees no need for " Zander " or other pendulum apparatus. Only experience can settle this question. But other competent authorities hold that the slow and rhythmical pendulum movements, in which the patient's own muscles day by day are more and more engaged, like rhythmical electrical contractions, approach very nearly to the natural movement and are therefore to be commended.

We believe that massage and other forms of physical treatment closely resemble one another in their actions on the body, mutually assist one another, and can usually be combined and associated with much advantage in the treatment especially of wounded soldiers. We may therefore hope that the time is approaching when massage and the use of remedial baths (or hydrological treatment) and medical, electrical, and mechanical treatment will be studied and practised, not as isolated and independent methods of treatment, but as a closely allied group of physical remedies. R. F. F.

